# Cerebral microbleeds and intracranial haemorrhage risk in patients anticoagulated for atrial fibrillation after acute ischaemic stroke or transient ischaemic attack (CROMIS-2): a multicentre observational cohort study

**DOI:** 10.1016/S1474-4422(18)30145-5

**Published:** 2018-06

**Authors:** Duncan Wilson, Gareth Ambler, Clare Shakeshaft, Martin M Brown, Andreas Charidimou, Rustam Al-Shahi Salman, Gregory Y H Lip, Hannah Cohen, Gargi Banerjee, Henry Houlden, Mark J White, Tarek A Yousry, Kirsty Harkness, Enrico Flossmann, Nigel Smyth, Louise J Shaw, Elizabeth Warburton, Keith W Muir, Hans Rolf Jäger, David J Werring, CROMIS-2 collaborators, John Aeron-Thomas, Prasanna Aghoram, Elaine Amis, Peter Anderton, Sreeman Andole, Ijaz Anwar, John Bamford, Azra Banaras, Aian Barry, Ruth Bellfied, Aienne Benford, Ajay Bhalla, Maneesh Bhargava, Biju Bhaskaran, Neelima Bhupathiraju, Jonathan Birns, Aian Blight, Angie Bowring, Ellen Brown, David Bruce, Amanda Buck, Kerry Bunworth, Ilse Burger, Laura Burgess, Mathew Burn, Evelyn Burssens, Mauian Burton, Nicola Butler, Denise Button, Michael Carpenter, Dinesh Chadha, Kausik Chatterjee, Lillian Choy, David Cohen, Lynne Connell, Martin Cooper, John Corrigan, Donna Cotterill, Gillian Courtauld, Susan Crawford, Claire Cullen, Krishna Dani, Amelia Daniel, Prabel Datta, Michelle Davis, Nicola Day, Mandy Doherty, Catherine Douglas, Karen Dunne, Collette Edwards, Charlotte Eglinton, Abduelbaset Elmarimi, Hedley Emsley, Timothy England, Daniel Epstein, Renuka Erande, Bernard Esisi, Rachel Evans, Pamela Farren, Pauline Fitzell, Glyn Fletcher, Rachel Gallifent, Rachel Gascoyne, Elio Giallombardo, Bindu Gregary, Gunaratam Gunathilagan, Paul Guyler, Brigid Hairsine, Michael Haley, Anne Hardwick, David Hargroves, Frances Harrington, Amanda Hedstrom, Clare Holmes, Senussi Hussein, Tanya Ingram, Sissi Ispoglou, Liz Iveson, Venetia Johnson, Frances Justin, Shahid Kausar, Karen Kee, Michael Keeling, Shagufta Khan, Agnieszka Kieliszkowska, Hayley Kingwell, Vinodh Krishnamurthy, Sagal Kullane, Balakrishna Kumar, Simon Leach, Sana Leason, Paula Lopez, Robert Luder, Barbara Madigan, Stuart Maguire, Holly Maguire, Karim Mahawish, Linetty Makawa, Maam Mamun, Dulka Manawadu, David Mangion, Aravindakshan Manoj, Syed Mansoor, Tracy Marsden, Rachel Marsh, Sheila Mashate, Michael McCormick, Clare McGolick, Madeleine McKee, Emma Mckenzie, Sanjeevikumar Meenakishundaram, Zoe Mellor, Amulya Misra, Amit Mistri, Azlisham Mohd Nor, Mushiya Mpelembue, Peter Murphy, Arumug Nallasivam, Ann Needle, Vinh Nguyen, Janice O'Connell, Paul O'Mahony, James Okwera, Chukwuka Orefo, Peter Owusu-Agyei, Anthea Parry, Adrian Parry-Jones, Kath Pasco, Chris Patterson, Cassilda Peixoto, Jane Perez, Nicola Persad, Mia Porteous, Michael Power, Christopher Price, Harald Proschel, Shuja Punekar, Janet Putterill, Marc Randall, Ozlem Redjep, Habib Rehman, Emma Richards, Victoria Riddell, Christine Roffe, Gill Rogers, Anthony Rudd, Kari Saastamoinen, Mahmud Sajid, Banher Sandhu, Christine Schofield, Jon Scott, Lakshmanan Sekaran, Pankaj Sharma, Jagdish Sharma, Simon Sharpe, Matthew Smith, Anew Smith, Nikola Sprigg, Julie Staals, Amy Steele, Gail Storey, Kelley Storey, Santhosh Subramonian, Jane Sword, Grainne Tallon, Garryck Tan, Margaret Tate, Jennifer Teke, Natalie Temple, Teresa Thompson, Sharon Tysoe, Djamil Vahidassr, Anouk van der Kwaak, Roland Veltkamp, Deborah Walstow, Caroline Watchurst, Fran Watson, Dean Waugh, Peter Wilkinson, David Wilson, Sarah Wilson-Owen, Belinda Wroath, Inez Wynter, Emma Young

**Affiliations:** a Stroke Research Centre, Department of Brain Repair and Rehabilitation, UCL Institute of Neurology and the National Hospital for Neurology and Neurosurgery, London, UK; b Department of Molecular Neuroscience, UCL Institute of Neurology and the National Hospital for Neurology and Neurosurgery, London, UK; c Lysholm Department of Neuroradiology and the Neuroradiological Academic Unit, Department of Brain Repair and Rehabilitation, UCL Institute of Neurology and the National Hospital for Neurology and Neurosurgery, London, UK; d Department of Statistical Science, University College London, London, UK; e Haemostasis Research Unit, Department of Haematology, University College London, London, UK; f Hemorrhagic Stroke Research Program, Department of Neurology, Massachusetts General Hospital Stroke Research Center, Harvard Medical School, Boston, MA, USA; g Centre for Clinical Brain Sciences, School of Clinical Sciences, University of Edinburgh, Edinburgh, UK; h Institute of Cardiovascular Sciences, University of Birmingham, Birmingham, UK; i Department of Neurology, Royal Hallamshire Hospital, Sheffield Teaching Hospitals Foundation Trust, Sheffield, UK; j Department of Neurology, Royal Berkshire Hospital, Reading, UK; k Department of Medicine, Royal Hampshire County Hospital, Winchester, UK; l Department of Medicine, Royal United Hospital, Bath, UK; m Department of Clinical Neurosciences, Addenbrookes Hospital, Cambridge, UK; n Institute of Neuroscience & Psychology, University of Glasgow, Queen Elizabeth University Hospital, Glasgow, UK

## Abstract

**Background:**

Cerebral microbleeds are a potential neuroimaging biomarker of cerebral small vessel diseases that are prone to intracranial bleeding. We aimed to determine whether presence of cerebral microbleeds can identify patients at high risk of symptomatic intracranial haemorrhage when anticoagulated for atrial fibrillation after recent ischaemic stroke or transient ischaemic attack.

**Methods:**

Our observational, multicentre, prospective inception cohort study recruited adults aged 18 years or older from 79 hospitals in the UK and one in the Netherlands with atrial fibrillation and recent acute ischaemic stroke or transient ischaemic attack, treated with a vitamin K antagonist or direct oral anticoagulant, and followed up for 24 months using general practitioner and patient postal questionnaires, telephone interviews, hospital visits, and National Health Service digital data on hospital admissions or death. We excluded patients if they could not undergo MRI, had a definite contraindication to anticoagulation, or had previously received therapeutic anticoagulation. The primary outcome was symptomatic intracranial haemorrhage occurring at any time before the final follow-up at 24 months. The log-rank test was used to compare rates of intracranial haemorrhage between those with and without cerebral microbleeds. We developed two prediction models using Cox regression: first, including all predictors associated with intracranial haemorrhage at the 20% level in univariable analysis; and second, including cerebral microbleed presence and HAS-BLED score. We then compared these with the HAS-BLED score alone. This study is registered with ClinicalTrials.gov, number NCT02513316.

**Findings:**

Between Aug 4, 2011, and July 31, 2015, we recruited 1490 participants of whom follow-up data were available for 1447 (97%), over a mean period of 850 days (SD 373; 3366 patient-years). The symptomatic intracranial haemorrhage rate in patients with cerebral microbleeds was 9·8 per 1000 patient-years (95% CI 4·0–20·3) compared with 2·6 per 1000 patient-years (95% CI 1·1–5·4) in those without cerebral microbleeds (adjusted hazard ratio 3·67, 95% CI 1·27–10·60). Compared with the HAS-BLED score alone (C-index 0·41, 95% CI 0·29–0·53), models including cerebral microbleeds and HAS-BLED (0·66, 0·53–0·80) and cerebral microbleeds, diabetes, anticoagulant type, and HAS-BLED (0·74, 0·60–0·88) predicted symptomatic intracranial haemorrhage significantly better (difference in C-index 0·25, 95% CI 0·07–0·43, p=0·0065; and 0·33, 0·14–0·51, p=0·00059, respectively).

**Interpretation:**

In patients with atrial fibrillation anticoagulated after recent ischaemic stroke or transient ischaemic attack, cerebral microbleed presence is independently associated with symptomatic intracranial haemorrhage risk and could be used to inform anticoagulation decisions. Large-scale collaborative observational cohort analyses are needed to refine and validate intracranial haemorrhage risk scores incorporating cerebral microbleeds to identify patients at risk of net harm from oral anticoagulation.

**Funding:**

The Stroke Association and the British Heart Foundation.

## Introduction

Atrial fibrillation increases the risk of ischaemic stroke by five times.^1^ In most individuals, oral anticoagulation with either vitamin K antagonists (VKAs) or direct oral anticoagulants (DOACs) is indicated because they reduce the risk of ischaemic stroke by about two thirds, with only a minimal increase in extracranial haemorrhage.^2^, ^3^ However, a devastating and unpredictable complication of oral anticoagulation is symptomatic intracranial haemorrhage, which has 42% in-hospital mortality and causes substantial disability in survivors.^4^ There is an unmet clinical need to reliably predict the risk of intracranial haemorrhage and to differentiate this from the risk of ischaemic stroke, to allow clinicians to assess the likely net clinical benefit of oral anticoagulation. Risk scores including clinical factors (eg, hypertension and age) have been developed to identify patients at high risk of bleeding on anticoagulation—including the HAS-BLED,^5^ HAEMORR_2_HAGES,^6^ and ATRIA^7^ scores—but these are of limited value in clinical decision making because they do not differentiate between prediction of ischaemic stroke and of intracranial haemorrhage.

**Research in context**

**Evidence before this study**

We searched MEDLINE without language restrictions for publications regarding cerebral microbleeds, atrial fibrillation, and ischaemic stroke published from inception up to Nov 3, 2017. We used the search terms (Cerebral microbleed*.mp OR microbleed.mp) AND (atrial fibrillation/ OR anticoagula*.mp OR anticoagula* OR warfarin.mp OR rivaroxaban.mp OR apixaban.mp OR edoxaban.mp OR dabigatran.mp) AND (cerebral infarction/ OR brain ischemia/ or stroke/ or isch?emi*.mp/ or transient isch?emic attack). We found four published prospective studies that reported rates of intracerebral haemorrhage in relation to baseline cerebral microbleeds in patients with ischaemic stroke or transient ischaemic attack treated with anticoagulation for atrial fibrillation. The largest study, involving 550 patients from Korea, showed a significant association between cerebral microbleed presence and intracerebral haemorrhage after adjusting for age, sex, and previous haemorrhagic stroke (hazard ratio [HR] 3·8; 95% CI 1·1–13·1), but none of the other studies were sufficiently powered to confirm this association. A post-hoc aggregate data meta-analysis of data mainly from small retrospective and prospective cohorts, with variable completeness and follow-up duration, suggested that cerebral microbleeds are associated with increased intracerebral haemorrhage risk, but could not adjust for confounding factors or develop risk models for intracranial haemorrhage.

**Added value of this study**

Our observational, predominantly UK-based, multicentre, prospective inception cohort study including 3366 patient-years of follow-up was designed and powered to determine whether cerebral microbleeds are independently associated with a higher risk of intracranial haemorrhage in patients with recent acute ischaemic stroke or transient ischaemic attack associated with atrial fibrillation and started for the first time on oral anticoagulation. We provide new evidence that in patients with ischaemic stroke or transient ischaemic attack and atrial fibrillation, cerebral microbleed presence is an independent risk factor for intracranial haemorrhage. We also show that the risk of intracranial haemorrhage increases as cerebral microbleed burden increases, but that the absolute event rate for ischaemic stroke remains higher than that of intracranial haemorrhage, even in patients with multiple cerebral microbleeds. We developed and internally validated a simple risk prediction score for intracranial haemorrhage, showing for the first time that the inclusion of cerebral microbleed presence as a neuroimaging biomarker improves the predictive value of a commonly used bleeding risk score based on clinical data alone (the HAS-BLED score).

**Implications of all the new evidence**

Our study provides proof of concept that including a neuroimaging biomarker (cerebral microbleeds) improves the predictive ability of clinical risk scores for intracranial haemorrhage—a potentially deadly complication of oral anticoagulation—which could help clinicians and patients to make better informed anticoagulation decisions. Our findings support further pooled meta-analyses of individual participant data from large prospective cohorts to increase the precision of risk estimates for intracranial haemorrhage, to determine whether high cerebral microbleed counts can identify patients who will experience net harm from oral anticoagulation, and to refine and validate intracranial haemorrhage risk scores incorporating clinical and neuroimaging factors including cerebral microbleeds.

Cerebral microbleeds are small, hypointense, round or ovoid areas identified on blood-sensitive MRI sequences (T2*-weighted gradient-recalled echo [GRE] or susceptibility-weighted imaging).^8^, ^9^ In most cases, cerebral microbleeds correspond pathologically to small clusters of haemosiderin-laden macrophages resulting from small self-limiting haemorrhages.^10^, ^11^ Thus, cerebral microbleeds are a promising radiological biomarker of the cerebral small vessel diseases that are prone to bleeding and cause most spontaneous intracerebral haemorrhages,^9^ so might be a specific and clinically useful predictor of anticoagulant-related intracranial haemorrhage. With the increasing use of blood-sensitive MRI, cerebral microbleeds can be detected in about 30% of patients with ischaemic stroke and atrial fibrillation,^12^ generating uncertainty about the risk–benefit balance of anticoagulation in patients with cerebral microbleeds.

We did an observational, prospective, multicentre, inception cohort study to determine whether cerebral microbleeds are independently associated with an increased risk of symptomatic intracranial haemorrhage in patients with recent acute ischaemic stroke or transient ischaemic attack with atrial fibrillation treated with anticoagulation. We developed and internally validated risk prediction scores for symptomatic intracranial haemorrhage including cerebral microbleed presence as a neuroimaging biomarker in addition to clinical risk factors.

## Methods

### Study design and participants

CROMIS-2 is an observational, multicentre, prospective, inception cohort study that recruited adults (ie, ≥18 years of age) with electrocardiogram-confirmed non-valvular atrial fibrillation who presented to one of 80 participating hospitals (79 in the UK and one in the Netherlands) with ischaemic stroke or transient ischaemic attack and were identified by their treating physician for anticoagulation treatment. We did not strictly control the timing of oral anticoagulation, which depended on best clinical judgment according to standard practice. We excluded patients if they could not undergo MRI, had a definite contraindication to anticoagulation, or had previously received therapeutic anticoagulation.

CROMIS-2 was approved by the UK National Health Service Research Ethics Committee. Patients with capacity gave informed written consent. When patients could not consent, we obtained written informed consent from a proxy as defined by relevant local legislation.

### Procedures

All patients underwent baseline brain MRI according to a predefined protocol parameter range, designed to detect relevant markers of cerebrovascular disease^13^ (see appendix), which required T2*-weighted GRE (echo time 10–45 ms), axial T1-weighted, axial T2-weighted, coronal fluid-attenuated inversion recovery, and diffusion-weighted imaging with apparent diffusion coefficient maps. MRIs were analysed for markers of cerebral small vessel disease defined according to consensus definitions^9^ using validated scales where available. We used the Microbleed Anatomical Rating Scale^14^ to identify and classify cerebral microbleeds as lobar or non-lobar (ie, deep, including the basal ganglia, thalamus, deep white matter, brainstem, and cerebellum). We rated white matter hyperintensities using the Fazekas and age-related white matter changes (ARWMC) scales,^15^, ^16^ and defined cortical superficial siderosis using consensus criteria.^17^, ^18^, ^19^ All neuroimaging ratings were done by a clinical research fellow (DW) trained by a professor of neuroradiology with cerebrovascular expertise (HRJ). A second trained clinical research fellow (GB) rated a random 10% sample for cerebral microbleed presence; we quantified intra-rater and inter-rater reliability for cerebral microbleed presence using Cohen's κ coefficient.

We obtained screening logs to assess selection bias. We obtained detailed clinical and demographic baseline data. From these data we calculated CHA₂DS₂VASc and HAS-BLED scores, designed to predict the risks of ischaemic stroke and major bleeding, respectively, in patients with non-valvular atrial fibrillation. We obtained follow-up information from patients and general practitioners at 6 months, 12 months, and 24 months via standardised structured postal questionnaires or telephone interviews. We obtained National Health Service digital data regarding hospital admissions or death during follow-up. For reported outcome events, we obtained additional clinical and radiological details from treating clinical teams and medical records to allow central adjudication, blinded to baseline neuroimaging findings.

### Outcomes

The primary outcome was symptomatic intracranial haemorrhage, defined as brain-imaging evidence of non-traumatic spontaneous intracranial haemorrhage with appropriate clinical symptoms, at any time before the final follow-up at 24 months. The secondary outcomes were recurrent ischaemic stroke and death of any cause. Further secondary outcomes not reported in this paper were transient ischaemic attack, cardiac ischaemic events (defined by dynamic electrocardiogram changes or troponin rise), subdivisions of intracranial haemorrhage (intracerebral [reported], subarachnoid, subdural, and extradural haemorrhage), major bleeding (defined as intracranial bleeding or extracranial bleeding in either a critical area or requiring hospitalisation and two units of blood transfusion^20^), quality of life, and long-term physical disability. A composite outcome of death, ischaemic stroke, and symptomatic inracranial haemorrhage was prespecified by the Steering Committee prior to the end of recruitment and data analysis, but was not prespecified in the statistical analysis plan.

Two professors of vascular neurology (DJW and MMB) and a clinical research fellow (DW) adjudicated all primary outcome events. A trained clinical research fellow (DW) adjudicated all ischaemic stroke outcomes; a random 10% of these were adjudicated by a professor of vascular neurology (DJW) and a professor of neuroradiology (HRJ). All adjudication was blinded to baseline cerebral microbleed ratings. In cases of disagreement, we reached consensus after discussion.

### Statistical analysis

We followed a prespecified published statistical analysis plan, which is provided in full in the appendix. We calculated a planned sample size of 1425 participants to detect a relative risk of 4·0 for intracranial haemorrhage associated with cerebral microbleeds, assuming an annual incidence of intracranial haemorrhage of 1·25% in those without cerebral microbleeds and that 20% of our population would have cerebral microbleeds; these estimates were derived from previous smaller studies.^13^ We included patients in the final analysis if they had undergone MRI with T2*-weighted GRE sequences of adequate technical quality to rate cerebral microbleeds.

We compared baseline demographics and risk factor profiles between those with and without cerebral microbleeds, and between those with and without our primary outcome event (symptomatic intracranial haemorrhage). We used appropriate statistical measures for categorical and continuous measures. We visually inspected the distributions of continuous variables using histograms, summarised as means with SDs or medians with IQRs. Groups were compared using the Mann-Whitney *U* test if not normally distributed or the *t* test if normally distributed; categorical variables were compared between groups with the χ^2^ test or, where appropriate, Fisher's exact test. Univariate Kaplan-Meier survival probabilities were estimated for those with and without cerebral microbleeds; we used the log-rank test to compare groups. We did univariable and multivariable Cox regression (adjusted for age and history of hypertension, as documented in our statistical analysis plan). We did three further multivariable Cox regression sensitivity analyses: first, including variables strongly associated with intracranial haemorrhage in univariate analysis; second, including cerebral microbleed presence and the HAS-BLED clinical bleeding risk score;^21^ and third, including cerebral microbleed presence and other neuroimaging markers of small vessel disease. We assessed the proportional hazards assumption through visual inspection of log-log plots of the log cumulative hazard against log time. We calculated absolute event rates per 1000 patient-years for the primary and the main secondary outcomes. For recurrent ischaemic stroke multivariable analysis, we adjusted for variables that differed between those with and without recurrent ischaemic stroke at the 20% level.

We developed two prediction models using Cox regression: first, including all predictors associated with intracranial haemorrhage at the 20% level in univariable analysis; and second, including cerebral microbleed presence and HAS-BLED score. We assessed calibration using the Cox calibration slopes, and quantified discrimination using Harrell's C-index. For bootstrapping validation, the models were re-fitted in 1000 bootstrap samples and applied to the original dataset. For each model, we then calculated the calibration slope and optimism-adjusted C-index.^22^ We also fitted these models using the lasso method^23^ to investigate possible overfitting. We did all statistical analysis using Stata version 12.0.

This study is registered with ClinicalTrials.gov, number NCT02513316.

### Role of the funding source

Neither the funders nor the sponsor had input into study design; data collection, data analysis, data interpretation; writing of the report; or the decision to submit the paper for publication. The corresponding author had full access to all the data in the study and had final responsibility for the decision to submit for publication.

## Results

**Figure 1:**
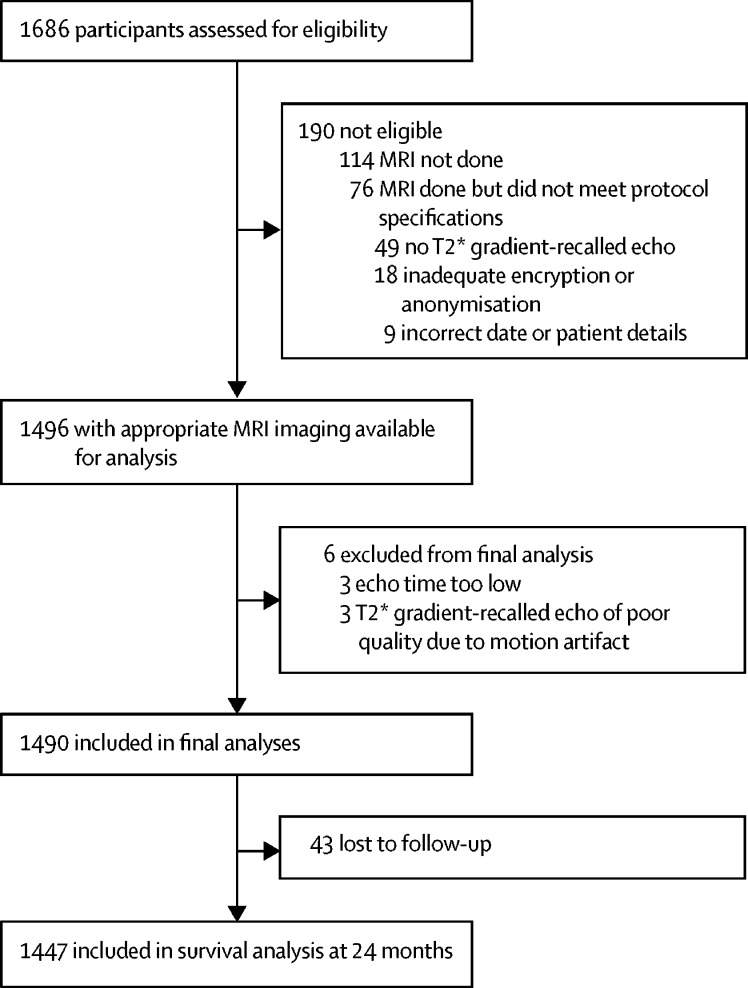
Participant flow

Between Aug 3, 2011, and July 31, 2015, 1686 potentially eligible patients consented from 79 centres across the UK and one centre in the Netherlands. After neuroimaging quality assurance, our final analysis included 1490 participants (1294 [87%] with 1·5 Tesla and 196 [13%] with 3 Tesla MRI scans); patient flow through the study is shown in figure 1. We found no significant differences in demographics, stroke risk factors, or stroke severity between patients included in the final analysis compared with those who gave consent and were screened but were ineligible or excluded. We collected screening logs from 26 sites (1120 patients) to assess selection bias; compared with the 506 patients who were included in the final analysis, the 614 patients who were eligible but did not consent were older (mean age 80 years [SD 11] *vs* 75 years [10]; p<0·0001), more likely to be female (252 [55%] of 460 patients *vs* 213 [42%] of 506 patients; p<0·0001), and had more severe strokes (median baseline National Institutes of Health Stroke Score 8 [IQR 3–16] *vs* 5 [2–10], p<0·0001).

Of 1490 patients included, 1447 (97%) had follow-up information available from any time during the 24 months (figure 1). The 43 patients without follow-up did not differ from those with follow-up in age (76 years [SD 10] *vs* 73 years [11]; p=0·166), hypertension (23 [58%] of 40 patients *vs* 907 [64%] of 1427 patients; p=0·43), or cerebral microbleed prevalence (seven [16%] of 43 patients *vs* 304 [21%] of 1447 patients; p=0·452).

Cerebral microbleeds were present in 311 (21%) of 1490 participants included in the final analyses, with a median cerebral microbleed count of one (IQR 1–3; table 1). Intra-rater and inter-rater reliability for the presence of cerebral microbleeds were excellent (intra-rater κ 0·93, 95% CI 0·86–1·00 and inter-rater 0·85, 0·74–0·96). Cerebral microbleeds were strictly lobar in 116 patients, strictly non-lobar (deep) in 120 patients, and mixed in 75 patients. 46 (3%) patients fulfilled the modified Boston criteria for cerebral amyloid angiopathy.^18^ Cortical superficial siderosis was identified in five patients, of which one was considered to be disseminated (table 1). 432 (29%) patients had severe white matter hyperintensities (ARWMC score^15^ ≥2 in either basal ganglia or white matter regions).

**Table 1:** Baseline characteristics

		**All patients (n=1490)**	**Patients with cerebral microbleeds (n=311)**	**Patients without cerebral microbleeds (n=1179)**
Age, years		76 (10)	78 (10)	75 (10)
Sex				
	Female	631 (42%)	129 (41%)	502 (43%)
	Male	859 (58%)	182 (59%)	677 (57%)
Hypertension		930/1467 (63%)	212/303 (70%)	718/1164 (62%)
Hyperlipidaemia		661/1469 (45%)	145/307 (47%)	516/1162 (44%)
Diabetes		251 (17%)	55 (18%)	196 (17%)
Ischaemic heart disease		243 (16%)	66 (21%)	177 (15%)
Previous ischaemic stroke		142 (10%)	41 (13%)	101 (9%)
Previous intracerebral haemorrhage		8 (1%)	3 (1%)	5 (<1%)
Alcohol use				
	Units per week	2 (0–9)	2 (0–7)	2 (0–10)
	>14 units per week	213/1384 (15%)	43 (15%)	170/1092 (16%)
Congestive heart failure		60 (4%)	20 (6%)	40 (3%)
Abnormal renal function		174 (12%)	46 (15%)	128 (11%)
Ethnicity				
	White	1414 (95%)	290 (95%)	1124 (95%)
	Asian*	33 (2%)	10 (3%)	23 (2%)
	Black	20 (1%)	5 (2%)	15 (1%)
C-reactive protein, mg/L		4·6 (2·0–12·0)	4·4 (2·0–12·0)	4·9 (2·0–11·2)
Platelet count		221 (185–265)	221 (185–265)	222 (183–265)
HAS-BLED score		3 (2–3)	3 (2–4)	3 (3–4)
CHA_2_DS_2_VASc score		5 (4–6)	5 (4–6)	5 (4–6)
Anticoagulation started		1436 (96%)	300 (96%)	1136 (96%)
Anticoagulant used				
	DOAC	542/1436 (37%)	121/300 (40%)	421/1136 (37%)
	VKA	894/1436 (62%)	179/300 (60%)	715/1136 (63%)
Concurrent antiplatelet use		57/894 (6%)	9 (3%)	48 (4%)
Poor time in therapeutic range†		133/894 (15%)	24/179 (13%)	109/715 (15%)
Anticoagulation stopped during follow-up		55/1436 (4%)	13/300 (4%)	42/1136 (4%)
Total white matter hyperintensity (ARWMC) score		1 (0–3)	2 (1–4)	1 (0–3)
Cerebral microbleeds		..	1 (1–3);range 1–107	NA
cSS presence		5 (<1%)	1 (<1%)	4 (<1%)

Data are n (%), n/N (%), mean (SD), or median (IQR). DOAC=direct oral anticoagulant; cSS=cortical superficial siderosis. ARWMC=age-related white matter changes. NA=not applicable. VKA=vitamin K antagonist.

* Asian denotes Indian, Pakistani, Bangladeshi, or “any other Asian background”.

† Poor time in therapeutic range for VKA use was defined as <60%.

The 1447 patients with follow-up data available provided 3366 patient-years of follow-up data (mean follow-up 850 days, SD 373). In this population, there were 14 symptomatic intracranial haemorrhages: 11 intracerebral haemorrhages, two subdural haemorrhages, and one subarachnoid haemorrhage. Compared with those who remained free of intracranial haemorrhage, patients who had a symptomatic intracranial haemorrhage during follow-up had a higher prevalence of diabetes, were more likely to have been treated with a VKA than a DOAC, and more likely to have cerebral microbleeds and cortical superficial siderosis (table 2). In the seven patients with a documented international normalised ratio at the time of the intracranial haemorrhage, the median international normalised ratio was 1·9 (IQR 1·4–4·0, range 1·1–4·8).

**Table 2:** Characteristics of patients with and without symptomatic intracranial haemorrhage at follow-up

			**Patients with symptomatic intracranial haemorrhage (n=14)**	**Patients without symptomatic intracranial haemorrhage (n=1433)**	**p value**
Age, years			79 (10)	76 (10)	0·322
Sex			..	..	0·620
	Female		5 (36%)	606 (42%)	..
	Male		9 (64%)	827 (58%)	..
Hypertension			8 (57%)	898/1411 (64%)	0·615
Hyperlipidaemia			8 (57%)	629/1413 (45%)	0·344
Diabetes			6 (43%)	236 (16%)	0·0086
Ischaemic heart disease			1 (7%)	238 (17%)	0·343
Previous ischaemic stroke			2 (14%)	138 (10%)	0·500
Previous intracerebral haemorrhage			0	8 (1%)	1·0
Alcohol use					
	Units per week		1·5 (0·0–5·0)	2 (0–9)	0·515
	>14 units per week		1/12 (8%)	205/1339 (15%)	0·496
Congestive heart failure			0	59 (4%)	0·440
Abnormal renal function			2 (14%)	169 (12%)	0·774
Ethnicity					
	White		14 (100%)	1356 (95%)	..
	Non-white		0	46 (3%)	0·492
		Asian*	0	29 (2%)	..
		Black	0	17 (1%)	..
C-reactive protein, mg/L			5·5 (4·6–16·2)	4·4 (2·0–12·0)	0·113
Platelet count			212 (167–225)	220 (185–264)	0·252
CHA_2_DS_2_VASc score			6 (4–6)	5 (4–6)	0·224
HAS-BLED score			2 (2–3)	3 (2–3)	0·144
Anticoagulation started			14 (100%)	1385 (97%)	0·485
Anticoagulant used			..	..	0·071
	DOAC		2 (14%)	523/1385 (38%)	..
	VKA		12 (86%)	862/1385 (62%)	..
Concurrent antiplatelets			1 (7%)	56 (4%)	0·536
Poor time in therapeutic range†			0	133/862 (15%)	0·145
Total white matter hyperintensity (ARWMC) score			1·5 (0·0–5·0)	1 (0·0–3·0)	0·968
Cerebral microbleed presence			7 (50%)	297 (21%)	0·0075
	Median		0·5 (0·0–3·0)	0 (0·0–0·0)	0·0034
	Range		0–12	0–107	NA
cSS presence			1 (7%)	4 (<1%)	<0·0001

Data are n (%), n/N (%), mean (SD), or median (IQR). Follow-up was at any time during the 24 months after enrolment, with a minimum of 6 months. DOAC=direct oral anticoagulant. cSS=cortical superficial siderosis. ARWMC=age-related white matter changes. NA=not applicable. VKA=vitamin K antagonist.

* Asian denotes Indian, Pakistani, Bangladeshi, or “any other Asian background”.

† Poor time in the therapeutic range for VKA use was defined as <60%.

The symptomatic intracranial haemorrhage event rate in patients with cerebral microbleeds was 9·8 per 1000 patient-years (95% CI 4·0–20·3) compared with 2·6 per 1000 patient-years (95% CI 1·1–5·4) in those without cerebral microbleeds; the absolute rate increase associated with cerebral microbleeds was 7·2 per 1000 patient-years (95% CI 2·9–14·9; table 3).

**Table 3:** Absolute event rates, absolute risks, and univariable and multivariable hazard ratios for symptomatic intracranial haemorrhage and recurrent ischaemic stroke during follow-up, according to baseline presence and burden of cerebral microbleeds

		Absolute event rate*	**Rate per 1000 patient-years (95% CI)**	**Absolute rate increase per 1000 patient-years (95% CI)**	**Univariable hazard ratio (95% CI)**	**Adjusted hazard ratio (95% CI)**†
**Symptomatic intracranial haemorrhage**						
No cerebral microbleeds		7/2654	2·6 (1·1 to 5·4)	1 (ref)	1 (ref)	1 (ref)
Cerebral microbleeds present		7/712	9·8 (4·0 to 20·3)	7·2 (2·9 to 14·9)	3·73 (1·31 to 10·64)	3·67 (1·27 to 10·60)
	1 cerebral microbleed	2/367	5·4 (0·7 to 19·7)	2·8 (−0·4 to 14·3)	2·04 (0·42 to 9·84)	2·03 (0·42 to 9·83)
	≥2 cerebral microbleeds	5/345	14·4 (4·7 to 33·8)	11·8 (3·6 to 28·4)	5·58 (1·77 to 17·58)	5·46 (1·70 to 17·51)
**Recurrent ischaemic stroke**						
No cerebral microbleeds		39/2608	15·0 (10·6 to 20·4)	1 (ref)	1 (ref)	1 (ref)
Cerebral microbleeds present		17/704	24·1 (14·1 to 38·7)	9·1 (3·5 to 18·3)	1·62 (0·92 to 2·87)	1·53 (0·85 to 2·76)
	1 cerebral microbleed	9/362	24·9 (11·4 to 47·2)	9·9 (0·8 to 32·2)	1·68 (0·82 to 3·47)	1·75 (0·84 to 3·65)
	≥2 cerebral microbleeds	8/341	23·4 (10·1 to 46·2)	8·4 (−0·5 to 25·8)	1·56 (0·73 to 3·35)	1·32 (0·60 to 2·93)

Data are calculated on the 1447 participants with follow-up data available.

* Calculated as number of events/patient-years.

† Adjusted for age and hypertension for symptomatic intracranial haemorrhage, and adjusted for age, sex, hypertension, diabetes, previous ischaemic stroke, and age-related white matter hyperintensities score for recurrent ischaemic stroke.

**Figure 2:**
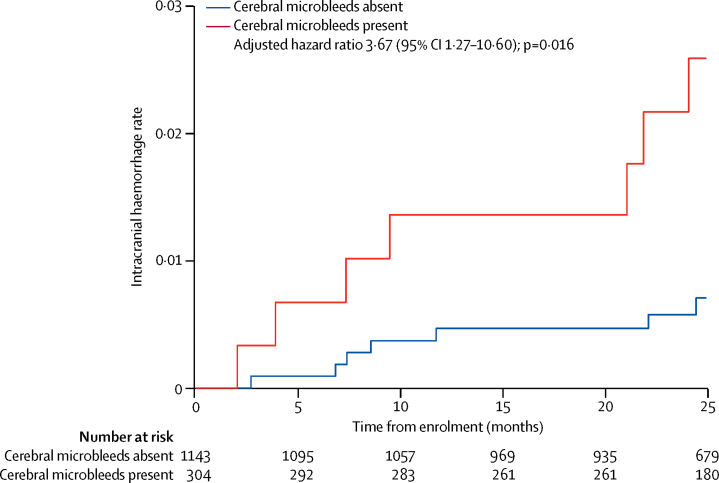
Probability of symptomatic intracranial haemorrhage according to the presence or absence of cerebral microbleeds The hazard ratio (HR) and 95% CI are derived from the model adjusted for hypertension and age.

Using the log-rank test for equality of survivor functions, we found that symptomatic intracranial haemorrhages were more frequent in patients with cerebral microbleeds compared with those without (p=0·0081). In univariable Cox regression, the hazard of symptomatic intracranial haemorrhage for patients with cerebral microbleeds was more than three times higher than that for patients without cerebral microbleeds; this risk was maintained in multivariable Cox regression analysis adjusted for hypertension and age (figure 2; table 3). The risk of symptomatic intracranial haemorrhage increased with increasing cerebral microbleed burden (overall p=0·017 from adjusted Cox regression for categories 0, 1, and ≥2 cerebral microbleeds and overall p=0·032 from unadjusted Cox regression for categories 0, 1, 2–4, and ≥5 cerebral microbleeds; table 3; appendix). We explored cerebral microbleed distribution and rates of symptomatic intracranial haemorrhage, but there were too few events within each category to draw reliable conclusions (appendix).

Of the 1490 patients recruited and identified to start anticoagulation, 1436 (96%) did so (table 1); 54 patients did not start because 12 had died, 13 refused or did not attend their anticoagulation clinic appointments, 17 had medical contraindications, and for 12 patients the reason was not specified. The median time from stroke symptoms until starting anticoagulation was 11 days (IQR 4–17); 894 (60%) of 1490 patients started a VKA and 542 (36%) of 1490 patients started a DOAC. Repeat analyses including only the 1436 anticoagulated participants did not affect our main result (univariable hazard ratio [HR] for cerebral microbleed presence 3·73; 95% CI 1·31–10·63). The type of anticoagulant (DOAC or VKA) did not significantly affect the hazard of symptomatic intracranial haemorrhage associated with cerebral microbleed presence (HR interaction term 0·88; 95% CI 0·04–17·13 p=0·92).

There were 56 recurrent ischaemic strokes during 3312 patient-years of follow-up. We observed an increased ischaemic stroke rate of 9·1 per 1000 patient-years (95% CI 3·5–18·3) associated with cerebral microbleeds (table 3). However, cerebral microbleed presence was not associated with recurrent ischaemic stroke in univariable or multivariable analyses (table 3; appendix). Mortality following symptomatic intracranial haemorrhage during follow-up was higher than that of recurrent ischaemic stroke (seven [50%, 95% CI 23–77] of 14 patients *vs* 12 [21%, 12–34] of 56 patients; p=0·041). In multivariable analysis, when adjusting for age and hypertension, cerebral microbleed presence was associated with symptomatic intracerebral haemorrhage (HR 4·24; 95% CI 1·27–14·08) but not death or the composite outcome (appendix).

In the first prediction model, we included variables that were significant at the 20% level in univariable analyses: cerebral microbleed presence, diabetes, DOAC use, and HAS-BLED score. We excluded cortical superficial siderosis owing to its rarity, and time in therapeutic range for VKA because it is captured within HAS-BLED. Missing alcohol scores for HAS-BLED were imputed using multiple imputation with chained equations^24^ (ten imputations). Fitting a model with all four predictors (cerebral microbleed presence, diabetes, DOAC use, and HAS-BLED score) produced an optimism-adjusted C-index of 0·74 (95% CI 0·60–0·88). In the second model, we included cerebral microbleed presence and HAS-BLED score (imputed as above), which produced an optimism-adjusted C-index of 0·66 (95% CI 0·53–0·80; see appendix for Cox calibration slopes). Compared with the HAS-BLED score alone (C-index 0·41; 95% CI 0·29–0·53), the first model (C-index difference 0·33, 0·14–0·51; p=0·00059) and the second model (C-index difference 0·25, 0·07–0·43; p=0·0065) were both better in predicting symptomatic intracranial haemorrhage.

We undertook three sensitivity analyses to confirm a robust independent association of cerebral microbleed presence with symptomatic intracranial haemorrhage. Because we observed only 14 symptomatic intracranial haemorrhages, we included a maximum of two predictor variables in each analysis. Cerebral microbleed presence remained an independent predictor of intracranial haemorrhage as follows: first, when adjusted for the two strongest univariable predictors (diabetes and anticoagulant type, but not cortical superficial siderosis because of its rarity): HR 3·63; 95% CI 1·27–10·38; second, when adjusted for HAS-BLED score: 5·64, 1·79–17·80; and third, when adjusted for other neuroimaging markers of small vessel disease: HR adjusted for total age-related white matter hyperintensities score 3·69, 1·26–10·74; HR adjusted for any cortical superficial siderosis 4·12, 1·42–11·97 (appendix). For each model, visual inspection of the log-log plots suggested that the proportional hazards assumption was satisfactory.

## Discussion

Our prospective, observational, multicentre cohort of patients anticoagulated after recent ischaemic stroke or transient ischaemic attack associated with atrial fibrillation shows that baseline cerebral microbleed presence is independently associated with an increased risk of symptomatic intracranial haemorrhage, but not of recurrent ischaemic stroke. However, the absolute rate of recurrent ischaemic stroke was much higher than the absolute rate of intracranial haemorrhage, even in those with cerebral microbleeds. We also show that the addition of a neuroimaging biomarker (cerebral microbleed presence) improves the predictive ability of a clinical bleeding risk score (HAS-BLED), which could help clinicians better identify patients at high risk of intracranial haemorrhage.

Our results are consistent with a smaller hospital-based cohort study in Korea of 550 patients with ischaemic stroke and atrial fibrillation^25^ that reported an increased risk of intracerebral haemorrhage associated with cerebral microbleeds (HR 3·8, 95% CI 1·1–13·1), as well as with a recent aggregate data meta-analysis.^12^ Our finding that diabetes is independently associated with symptomatic intracranial haemorrhage has not, to the best of our knowledge, been previously reported in ischaemic stroke cohorts. However, a large community-based study reported that diabetes was associated with intracerebral haemorrhage risk (1·59, 1·26–2·02),^26^ whereas another study^27^ of older patients (≥75 years of age; median age 82 years) with atrial fibrillation attending an anticoagulation clinic found an association between diabetes and major bleeding (mostly intracranial haemorrhage; odds ratio 4·4, 95% CI 1·3–14·7).

Our finding that cerebral microbleed presence was not associated with recurrent ischaemic stroke differs from our recent meta-analysis^28^ of patients with recent ischaemic stroke or transient ischaemic attack, probably because the meta-analysis included mostly patients without atrial fibrillation and treated with antiplatelet therapy. The association of cerebral microbleed presence with future symptomatic intracranial haemorrhage but not ischaemic stroke risk in our cohort supports the hypothesis that cerebral microbleeds are a neuroimaging biomarker of a bleeding-prone arteriopathy specifically relevant for intracranial haemorrhage associated with anticoagulation. However, the relationship between cerebral microbleed presence and recurrent ischaemic stroke risk, while not statistically significant, also favoured a positive association. Thus, cerebral microbleeds, as a marker of overall vascular fragility, might not reliably discriminate between intracranial bleeding and ischaemic stroke risks, but this important question requires further study. Indeed, the absolute event rate of ischaemic stroke in patients with cerebral microbleeds (24·1 per 1000 patient-years) was much higher than the absolute event rate of symptomatic intracranial haemorrhage (9·8 per 1000 patient-years). By contrast with cerebral microbleeds, white matter hyperintensities were not associated with symptomatic intracranial haemorrhage in our study, in keeping with data from two previous smaller similar cohort studies.^25^, ^29^

Although recent meta-analyses of ischaemic stroke and transient ischaemic attack cohorts have explored the risk of intracerebral haemorrhage in patients with five or more cerebral microbleeds,^12^, ^28^ we did not present hazard ratios for this subgroup because of the very low number of participants with high cerebral microbleed counts and of symptomatic intracranial haemorrhage events, which could lead to statistically unreliable results and over-interpretation. Thus, although we found that the rate of symptomatic intracranial haemorrhage increased as cerebral microbleed burden increased (and the rate of recurrent ischaemic stroke remained stable), we could not establish whether a cerebral microbleed burden threshold exists at which the absolute event rate of intracranial haemorrhage exceeds that of ischaemic stroke (ie, where anticoagulation might be associated with net harm as judged by absolute event rates). We found that having a single cerebral microbleed was not associated with a higher hazard of symptomatic intracranial haemorrhage, possibly because one cerebral microbleed reflects only minor small vessel disease, or because of limited inter-rater and intra-rater reliability for one cerebral microbleed.^14^, ^30^

Most currently available bleeding risk scores (which include clinical risk factors but not neuroimaging biomarkers) show only modest predictive value for intracranial haemorrhage with C-indexes of about 0·5,^31^ although a post-hoc analysis^32^ of the ROCKET-AF study suggested that including more detailed quantitative factors (eg, platelet count, albumin, diastolic blood pressure) might also improve the predictive performance. Our findings suggest that adding cerebral microbleed presence as a neuroimaging biomarker to a widely used clinical risk score (HAS-BLED) might improve specificity and sensitivity in identifying ischaemic stroke and transient ischaemic attack patients at high risk of intracranial haemorrhage; this knowledge should allow better informed counselling, closer follow-up of high-risk individuals, rational anticoagulant choice, consideration of non-anticoagulant treatment options (eg, left atrial appendage occlusion), and more aggressive management of modifiable risk factors for intracranial haemorrhage (eg, hypertension, anticoagulant monitoring, and compliance). Large-scale collaborations are required to refine and validate robust risk prediction scores. Meanwhile, our findings suggest that future risk scores to identify patients with stroke at risk of intracranial haemorrhage should include cerebral microbleeds as a neuroimaging biomarker in addition to clinical parameters.

Our study has some important strengths. We prospectively studied a large inception cohort of patients at multiple hospital stroke units using predefined MRI sequences, rated for neuroimaging markers of small vessel disease using validated scales by a single trained observer. We followed up 97% of our cohort, and experienced observers adjudicated all primary events blinded to baseline cerebral microbleed presence. We undertook survival analysis to account for baseline confounding factors and varying follow-up, and followed a prespecified statistical analysis plan.

We also acknowledge our study's limitations. Our cohort is likely to be affected by selection bias because patients with more severe strokes were less likely to be enrolled. Nevertheless, our cohort is likely to be representative of patients considered for anticoagulation soon after ischaemic stroke. All local investigators agreed to a policy of making anticoagulation decisions without considering cerebral microbleeds, but it was not possible to mandate and monitor blinding at the 79 participating centres. Although bias remains possible owing to the absence of formal blinding, 96% of all recruited patients with satisfactory MRI sequences were started on anticoagulants regardless of cerebral microbleed status. The proportion of DOAC use was similar in patients with cerebral microbleeds (40%) and without cerebral microbleeds (36%), suggesting that cerebral microbleeds did not influence the choice of VKA or DOAC. Because most participants in CROMIS-2 took VKA, our findings might not be generalisable to health-care settings where DOACs are the most widely used anticoagulant. Our study had a low rate of symptomatic intracranial haemorrhage, limiting our ability to adjust for multiple confounders, the robustness of our risk prediction models and, importantly, our ability to determine how increasing cerebral microbleed burden might relate to intracranial haemorrhage risk. Although we standardised parameters for MRI, different scanners with different magnetic field strengths were used, which can influence cerebral microbleed detection.^33^ Furthermore, T2*-weighted GRE MRI sequences are less sensitive to cerebral microbleeds than is susceptibility-weighted imaging,^34^ so our interpretation of cerebral microbleed-related risk might not generalise to susceptibility-weighted imaging data. Treatment decisions might be influenced by clinical nihilism about intracranial haemorrhage compared with ischaemic stroke; judgment of different apparent severities of incident intracranial haemorrhage compared with ischaemic stroke might in part be artefacts of clinical behaviour.

The low incidence of symptomatic intracranial haemorrhage in patients with ischaemic stroke or transient ischaemic attack anticoagulated for atrial fibrillation makes randomised controlled trials in this field challenging. However, large-scale international pooled collaborative observational cohort analyses should help to refine risk prediction and determine whether high cerebral microbleed counts might be associated with an increased risk of intracranial haemorrhage sufficient to clearly identify patients at risk of net harm from oral anticoagulation.
