# Corrections

**DOI:** 10.1016/S1474-4422(18)30205-9

**Published:** 2018-07

*Wilson D, Ambler G, Shakeshaft C, et al. Cerebral microbleeds and intracranial haemorrhage risk in patients anticoagulated for atrial fibrillation after acute ischaemic stroke or transient ischaemic attack (CROMIS-2): a multicentre observational cohort study.* Lancet Neurol *2018; **17**: 539–47*—The appendix of this Article has been corrected as of May 25, 2018.

