# Supplementary material for: Cerebral microbleeds and intracranial haemorrhage risk in patients anticoagulated for atrial fibrillation after acute ischaemic stroke or transient ischaemic attack (CROMIS-2): a multicentre observational cohort study
Source: Lancet Neurol. 2018 Jun;17(6):539–47. doi: 10.1016/S1474-4422(18)30145-5 (PMC5956310; doi:10.1016/S1474-4422(18)30145-5)
Supplement: Supplementary appendix [file mmc1.pdf]

THE LANCET
Neurology

Supplementary appendix

This appendix formed part of the original submission and has been peer reviewed. We post it as supplied by the authors.

Supplement to: Wilson D, Ambler G, Shakeshaft C, et al, on behalf of the CROMIS-2 collaborators. Cerebral microbleeds and intracranial haemorrhage risk in patients anticoagulated for atrial fibrillation after acute ischaemic stroke or transient ischaemic attack (CROMIS-2): a multicentre observational cohort study. *Lancet Neurol* 2018; published online May 16. http://dx.doi.org/10.1016/S1474-4422(18)30145-5.

Supplementary appendix

MRI protocol

The CROMIS-2 MRI protocol included axial T2-weighted, T2*-weighted gradient-recalled echo (GRE), diffusion-weighted imaging (diffusion weighted imaging and apparent diffusion coefficient maps), coronal T1-weighted, and FLAIR MR sequences.

T2*-weighted GRE with the following sequence parameters was essential for inclusion in the primary analysis:

Optimal
Slice thickness (ST) 3 mm
Slice gap (SG): ideally no gap
Echo time (TE): 20–30 ms

Acceptable
Slice thickness (ST) 3–5 mm
Slice gap (SG): not more than 1 mm
Echo time (TE): 10–40 ms

Field strength 1.5T or 3.0T are both acceptable

Supplementary table 1: CHA_2DS_2VASC and HAS-BLED scores in participants with CMBs compared to those without CMBs

CHA_2DS_2VASc score	CMBs present n (%) n=311	No CMBs present n (%) n=1179
2	12 (4)	58 (5)
3	26 (9)	160 (14)
4	51 (17)	245 (21)
5	77 (26)	297 (26)
6	87 (29)	269 (23)
7	38 (13)	99 (9)
8	8 (3)	15 (1)
9	1 (0·3)	3(0·3)

HAS-BLED	CMBs Present n (%) n=271	No CMBs present n (%) n=1033
1	4 (1)	10 (1)
2	24 (9)	127 (12)
3	79 (29)	348 (34)
4	91 (34)	331 (32)
5	62 (23)	180 (17)
6	10 (4)	33 (3)
7	1 (0.4)	4 (0·4)

Key:

CMB – cerebral microbleed

Supplementary table 2: CMB burden categories in patients with and without intracranial haemorrhage

**Variable**	**Total n (%)**	**Patients with symptomatic intracranial hemorrhage n =14**	**Patients without symptomatic intracranial hemorrhage n =1433**	**Hazard ratio value in univariate analysis (95% CI)**
**No CMBs n (%)**	1179 (79)	7 (50)	1172 (82%)	*reference*
**1 CMB n (%)**	159 (11)	2 (14)	157 (11)	2.04 (0.42 to 9.84)
**2-4 CMBs n (%)**	103 (7)	3 (21)	100 (7)	5.04 (1.30 to 19.50)
**≥ 5 CMBs n (%)**	49 (3)	2 (14)	47 (3)	6.64 (1.38 to 39.59)

Key:

CMB – cerebral microbleed

HR - hazard ratio

CI - confidence interval

Supplementary table 3: Absolute event rates, risk increase and hazard ratios for symptomatic intracranial haemorrhage and ischaemic stroke according to CMB distribution

All hazard ratios are adjusted for CMB number.

	**Symptomatic intracranial haemorrhage**			**Recurrent ischaemic stroke**		
	**Absolute event rate (n/patient-years)**	**Rate/1000 patient years (95% CI)**	**Hazard ratio (95% CI)**	**Absolute event rate (n/patient years)**	**Rate/1000 patient years (95% CI)**	**Hazard ratio (95% CI)**
**No CMBs**	7/2654	3 (1 to 5)	*Reference*	39/2608	15 (11 to 20)	*Reference*
**Strictly lobar CMBs**	3/243	12 (3 to 36)	3·31 (0·92 to 11·90)	4/243	16 (4 to 42)	0·94 (0·34 to 2·61)
**Strictly Deep CMBs**	1/285	0·3 (0·00 to 20)	0·83 (0·11 to 6·36)	8/278	29 (12 to 57)	1·82 (0·86 to 3·84)
**Mixed CMBs**	3/184	16 (3 to 48)	5·33 (1·23 to 23·07)	5/183	27 (9 to 64)	1·57 (0·55 to 4·50)
**Multiple strictly lobar CMBs**	1/91	11 (0·3 to 61)	2·45 (0·31 to 19.03)	2/89	22 (2 to 81)	1·20 (0·29 to 4·98)

Key: CMB cerebral microbleed; CI confidence interval

Supplementary table 4: Cox regression analysis for the primary outcome (symptomatic intracranial haemorrhage) including CMBs and the two other strongest predictors from univariable analysis

Variable	HR	95% CI	p value
**CMB presence**	3·63	1·27 to 10·38	0·016
**Diabetes**	3·49	1·21 to 10·10	0·021
**DOAC use**	0·31	0·07 to 1·38	0·123

Key:

CMB –cerebral microbleed

DOAC – direct oral anticoagulant

HR hazard ratio

CI confidence interval

Supplementary table 5: Association of brain imaging markers of cerebral small vessel disease with symptomatic intracranial haemorrhage in univariable analyses, and effects of adjusting for each imaging marker on the association of CMB presence and symptomatic intracranial haemorrhage

Variable	Definition of variable	Univariable Hazard Ratio for symptomatic intracranial haemorrhage (95% CI)	Hazard Ratio for CMB presence and symptomatic intracranial haemorrhage when each biomarker is entered as an 'adjustment variable'
**White matter hyperintensities**	Total ARWMC score	1·07<br>(0·86 to 1·34)	3·69<br>(1·26 to 10·74)
	Posterior predominant ARWMC	0·88<br>(0·20 to 3·94)	3·78<br>(1·32 to 10·79)
	ARWMC score dichotimised*	1·03<br>(0·32 to 3·29)	3·84<br>(1·33 to 11·10)
**cSS**	Any	24·78<br>(3·24 to 189·68)	4·12<br>(1·42 to 11·97)
	Disseminated**	N/A	3·73<br>(1·31 to 10·63)

Key:

CMB – cerebral microbleed

cSS – cortical superficial siderosis

ARWMC – age related white matter changes

* Defined as ARWMC score of 2 or above in either Basal ganglia or white matter regions

** Defined as siderosis affecting 3 or more non-contiguous cerebral sulci

Supplementary table 6: Characteristics of patients with and without recurrent ischaemic stroke

Variable	Ischaemic stroke events (n =56)	No ischaemic stroke events (n= 1391)	p value
**Age mean (SD)**	79 (10)	76 (10)	0·026
**Sex female n (%)**	32 (57)	579 (42)	0·021
**Hypertension n (%)**	42 (75)	864 (63)	0·070
**Hypercholesterolaemia n (%)**	24 (43)	613 (45)	0·784
**Diabetes mellitus n (%)**	17 (31)	225 (16)	0·004
**Previous ischaemic stroke n (%)**	9 (17)	131(10)	0·086
**Ischaemic heart disease**	9 (16)	230 (17)	0·927
**Previous intracerebral haemorrhage n (%) #**	1 (2)	7 (0·5)	0·203
**Alcohol units/ week median (IQR)**	1 (0 to 4)	2 (0 to 9)	0·062
**Alcohol use >14 units/week n (%)**	3 (5)	206 (16)	0·039
**CHA_2DS_2VASc score median (IQR)**	6 (5 to 7)	5 (4 to 6)	<0·0001
**HASBLED score median (IQR)**	3(2 to 3)	3 (2 to 3)	0·2
**Anticoagulation started n (%)**	54 (96)	1345 (97)	0·914
**DOAC used n (%)** **available in 1436 patients who started anticoagulation**	18 (33)	507 (38)	0·516
**Poor time in therapeutic range n (%)** *available in 717/874 of patients on VKA*	6 (11)	158 (11)	0·881
**ARWMC score median (IQR)**	2 (1 to 4)	1 (0 to 3)	0·012
**CMB presence n (%)**	17 (30)	287 (21)	0·08
**cSS presence n (%) #**	0 (0)	5 (0·4)	1.0

Key:

DOAC –non vitamin K antagonist oral anticoagulants

CMB –cerebral microbleed

cSS – cortical superficial siderosis

Poor therapeutic time in range for patients treated with vitamin K antagonists defined as <60%

ARWMC –age related white matter changes

Fisher's exact test

Supplementary table 7: Harrell's C-index and Cox calibration slope for the two prediction models. Model 1 includes CMB presence, diabetes, DOAC use and HAS-BLED score; model 2 includes CMB presence and HAS-BLED score

	Adjusted Harrell's C-index (95% CI)	Cox calibration slope (95% CI)
**Model 1**	0.74 (95% CI: 0.60 to 0.88)	0.92 (95% CI: 0.34 to 1.46)
**Model 2**	0.66 (95% CI: 0.53 to 0.80)	0.96 (95% CI: 0.19 to 1.72)

Note: Some bootstrap samples were omitted because of model instability (14% for model 1 and 2% for model 2)

Supplementary figure 1: Forest plots showing the incidence and hazard ratio with 95% confidence intervals of symptomatic intracranial haemorrhage and recurrent ischaemic stroke according to CMB burden

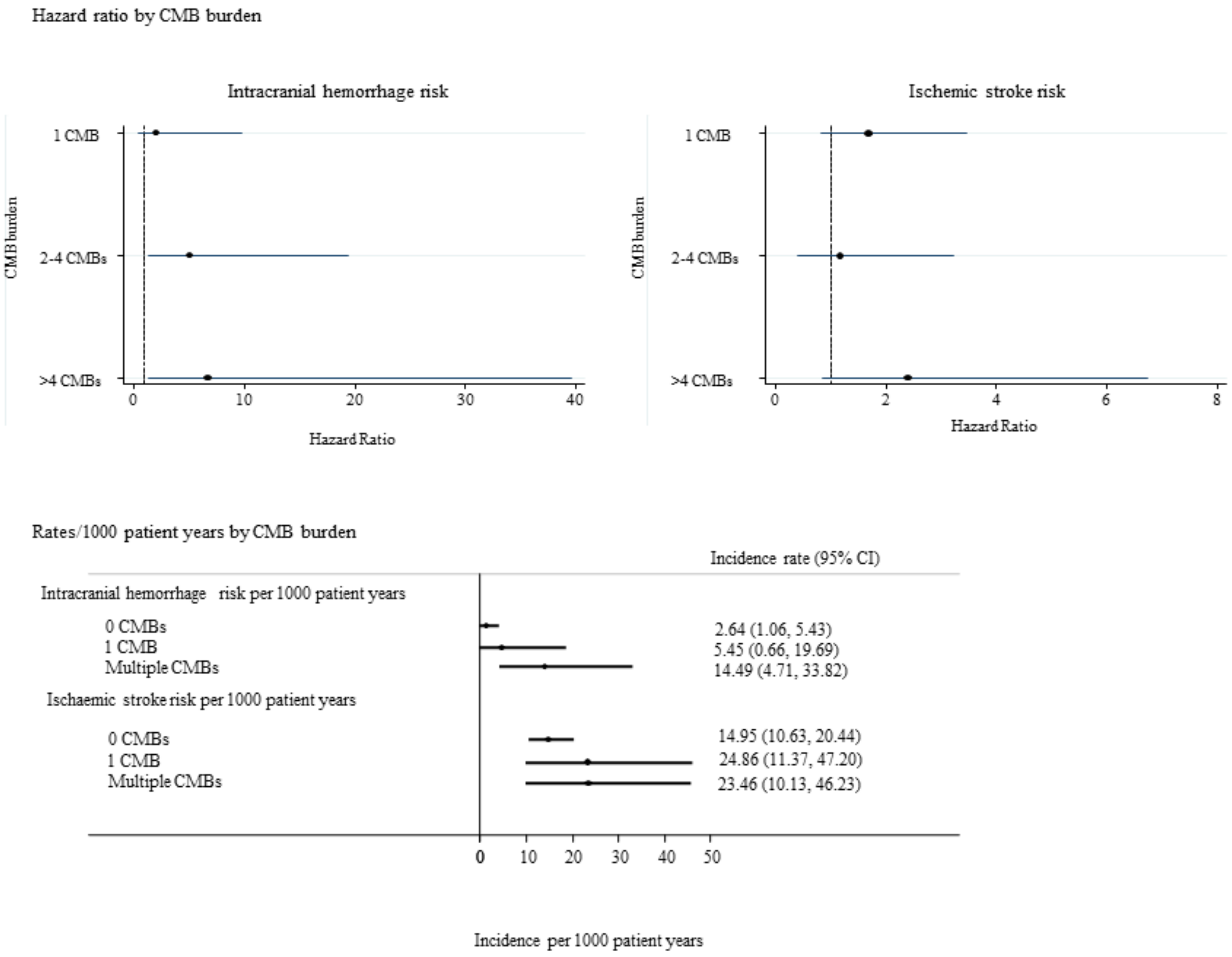

Supplementary figure 2: Forest plots of the hazard ratio for secondary outcomes in participants with CMBs vs. those without CMBs

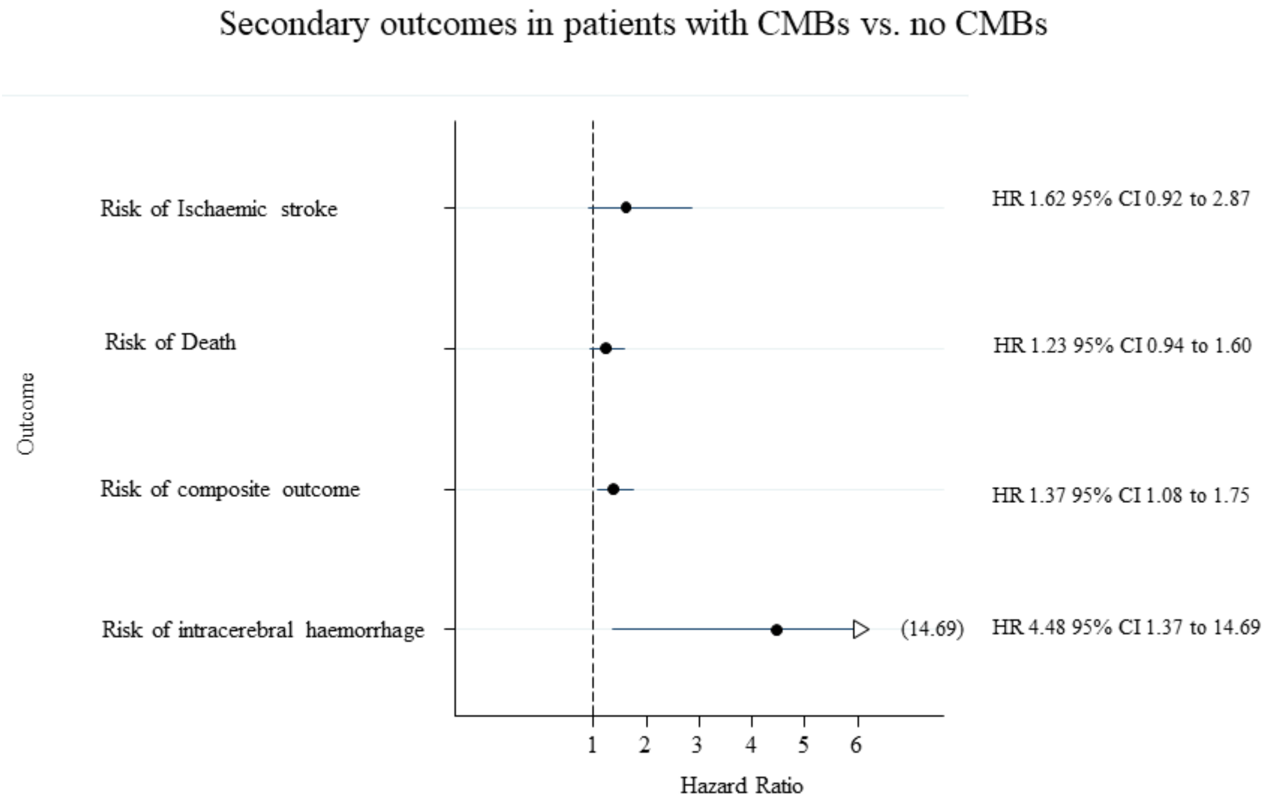

The composite outcome included symptomatic intracranial haemorrhage, ischaemic stroke or death.

The CROMIS-2 Collaborators

First name	Surname (in alphabetical order)
John	Aeron-Thomas
Prasanna	Aghoram
Elaine	Amis
Peter	Anderton
Sreeman	Andole
Ijaz	Anwar
John	Bamford
Azra	Banaras
Aian	Barry
Ruth	Bellfield
Aienne	Benford
Ajay	Bhalla
Maneesh	Bhargava
Biju	Bhaskaran
Neelima	Bhupathiraju
Ekaterina	Biggs
Jonathan	Birns
Adrian	Blight
Angie	Bowring
Ellen	Brown
David	Bruce
Amanda	Buck
Kerry	Bunworth
Ilse	Burger
Laura	Burgess
Mathew	Burn
Evelyn	Burssens
Maudrian	Burton
Nicola	Butler
Denise	Button
Sarah	Caine
Michael	Carpenter
Dinesh	Chadha
Kausik	Chatterjee
Lillian	Choy
David	Cohen
Bridget	Colam
Lynne	Connell
Martin	Cooper
John	Corrigan
Donna	Cotterill
Gillian	Courtauld
Linda	Cowie
John	Coyle
Susan	Crawford

Claire	Cullen
Krishna	Dani
Amelia	Daniel
Khaled	Darawil
Prabel	Datta
Michelle	Davis
Nicola	Day
Sharon	Dealing
Mandy	Doherty
Deborah	Dellafera
Catherine	Douglas
Karen	Dunne
Collette	Edwards
Charlotte	Eglinton
Abduelbaset	Elmarimi
Hedley	Emsley
Timothy	England
Daniel	Epstein
Renuka	Erande
Bernard	Esisi
Rachel	Evans
Pamela	Farren
Pauline	Fitzell
Glyn	Fletcher
Rachel	Gallifent
Rachel	Gascoyne
Elio	Giallombardo
Bindu	Gregary
Gunaratam	Gunathilagan
Paul	Guyler
Brigid	Hairsine
Michael	Haley
Anne	Hardwick
David	Hargroves
Frances	Harrington
Ahamad	Hassan
Amanda	Hedstrom
Melinda	Holden
Clare	Holmes
Senussi	Hussein
Tanya	Ingram
Sissi	Ispoglou
Liz	Iveson
Venetia	Johnson
Frances	Justin
Shahid	Kausar
Karen	Kee
Michael	Keeling

Deborah	Kelly
Shagufta	Khan
Agnieszka	Kieliszkowska
Hayley	Kingwell
Vinodh	Krishnamurthy
Sagal	Kullane
Balakrishna	Kumar
Geraldine	Landers
Enas	Lawrence
Simon	Leach
Sana	Leason
Paula	Lopez
Caroline	Lovelock
Robert	Luder
Barbara	Madigan
Stuart	Maguire
Holly	Maguire
Karim	Mahawish
Linetty	Makawa
Maam	Mamun
Dulka	Manawadu
David	Mangion
Aravindakshan	Manoj
Syed	Mansoor
Tracy	Marsden
Rachel	Marsh
Sheila	Mashate
Michael	McCormick
Clare	McGoldrick
Madeleine	McKee
Emma	Mckenzie
Sanjeevikumar	Meenakishundaram
Zoe	Mellor
Amulya	Misra
Amit	Mistri
Azlisham	Mohd Nor
Mushiya	Mpelembue
Peter	Murphy
Arumug	Nallasivam
Ann	Needle
Vinh	Nguyen
Janice	O'Connell
Paul	O’Mahony
James	Okwera
Chukwuka	Orefo
Peter	Owusu-Agyei
Anthea	Parry
Adrian	Parry-Jones

Kath	Pasco
Chris	Patterson
Cassilda	Peixoto
Jane	Perez
Nicola	Persad
Mia	Porteous
Michael	Power
Mathuri	Prabhakaran
Christopher	Price
Harald	Proschel
Shuja	Punekar
Janet	Putterill
Marc	Randall
Ozlem	Redjep
Habib	Rehman
Emma	Richards
Victoria	Riddell
Christine	Roffe
Gill	Rogers
Anthony	Rudd
Kari	Saastamoinen
Mahmud	Sajid
Banher	Sandhu
Christine	Schofield
Jon	Scott
Lakshmanan	Sekaran
Pankaj	Sharma
Jagdish	Sharma
Simon	Sharpe
Matthew	Smith
Andrew	Smith
Nikola	Sprigg
Julie	Staals
Amy	Steele
Gail	Storey
Kelley	Storey
Santhosh	Subramonian
Appu	Suman
Jane	Sword
Grainne	Tallon
Garryck	Tan
Margaret	Tate
Jennifer	Teke
Natalie	Temple
Teresa	Thompson
Sharon	Tysoe
Djamil	Vahidassr
Anouk	van der Kwaak

Roland	Veltkamp
Deborah	Walstow
Caroline	Watchurst
Fran	Watson
Dean	Waugh
Peter	Wilkinson
David	Wilson
Jennifer	Wilson
Sarah	Wilson-Owen
Belinda	Wroath
Inez	Wynter
Emma	Young

University College London Hospitals NHS Foundation Trust

Joint Research Office

Office Location:
1st Floor Maple House
149 Tottenham Court Road
London W1

Postal Address:
Rosenheim Wing, Ground Floor
25 Grafton Way
London, WC1E 5DB

STATISTICAL ANALYSIS PLAN

**Study Name**:	The Clinical Relevance of Microbleeds In Stroke Study (CROMIS-2): Sub-Study 1 (AF)
**Chief Investigator**:	David Werring
**Statisticians**:	Gareth Ambler
**Protocol Date**:	15 November 2016

Version History

0.4	Draft analysis plan	19/9/14
0.5	Analysis changed to survival	15/11/16

OVERVIEW OF STUDY

The following sections are taken from the protocol paper. The numbers in parentheses refer to references in the original paper.

BACKGROUND

Study I (AF) is an observational inception cohort study (n=1425) of patients started on oral anticoagulant (without prior use, anticoagulant use and timing dependant on treating physician) for presumed cardioembolic stroke due to non-valvular AF with follow-up for the occurrence of ICH, other vascular event including ischaemic stroke, for an average of two years. Our main baseline exposures (risk factors of interest) are the presence of CMBs and other markers of small vessel disease on MRI, and genetic polymorphisms in candidate genes with potential functional relevance to ICH risk.

OUTCOME MEASURES

Primary Outcome Measures

Symptomatic intracranial haemorrhage (including ICH) confirmed on brain imaging. Intracranial haemorrhage includes any bleeding within the skull, regardless of the site. We will record the incidence of different haemorrhage subtypes (intracerebral, intraventricular, subdural, extradural, and subarachnoid).

Secondary Outcome Measures

Ischaemic stroke, TIA, cardiac events (including myocardial infarction), death of any cause, subdivisions of intracranial haemorrhage (intracerebral, subarachnoid, subdural, extradural haemorrhage), any major haemorrhagic events other than ICH, quality of life and long term physical disability.

SAMPLE SIZE CALCULATIONS

Based on our sample size calculations we will recruit a total of 1425 patients from UK centres over 47 months. We expect that 20% of our cohort will have CMBs and that 2% will have an ICH within 2 years. If we assume a conservative relative risk of 4, smaller than the one found in the largest prospective data in an ischaemic stroke cohort investigated for CMBs published to date (i.e. 7.3) (28), then we would expect the rate of ICH at 2 years follow-up to be 5.0% in patients with CMBs, compared with 1.25% in patients without CMBs. This difference would be clinically important and would tip the risk-benefit judgement in favour of avoiding or reducing the intensity of oral anticoagulation, or substituting an antiplatelet agent in patients with CMBs. To detect such a difference as statistically significant at the 5% level with 90% power would require 1425 patients. The best current evidence for the relative risk associated with CMBs in Caucasian populations is 3.9 (33), so we have calculated the power for a range of risk ratios, with all other assumptions kept the same (Figure). The figure suggests that we would still have 80% power to detect a statistically significant effect if the true relative risk was as low as 3.3. Attrition will also reduce power. However, we will still have 80% power even if attrition was as large as 28% (based on a relative of risk of 4).

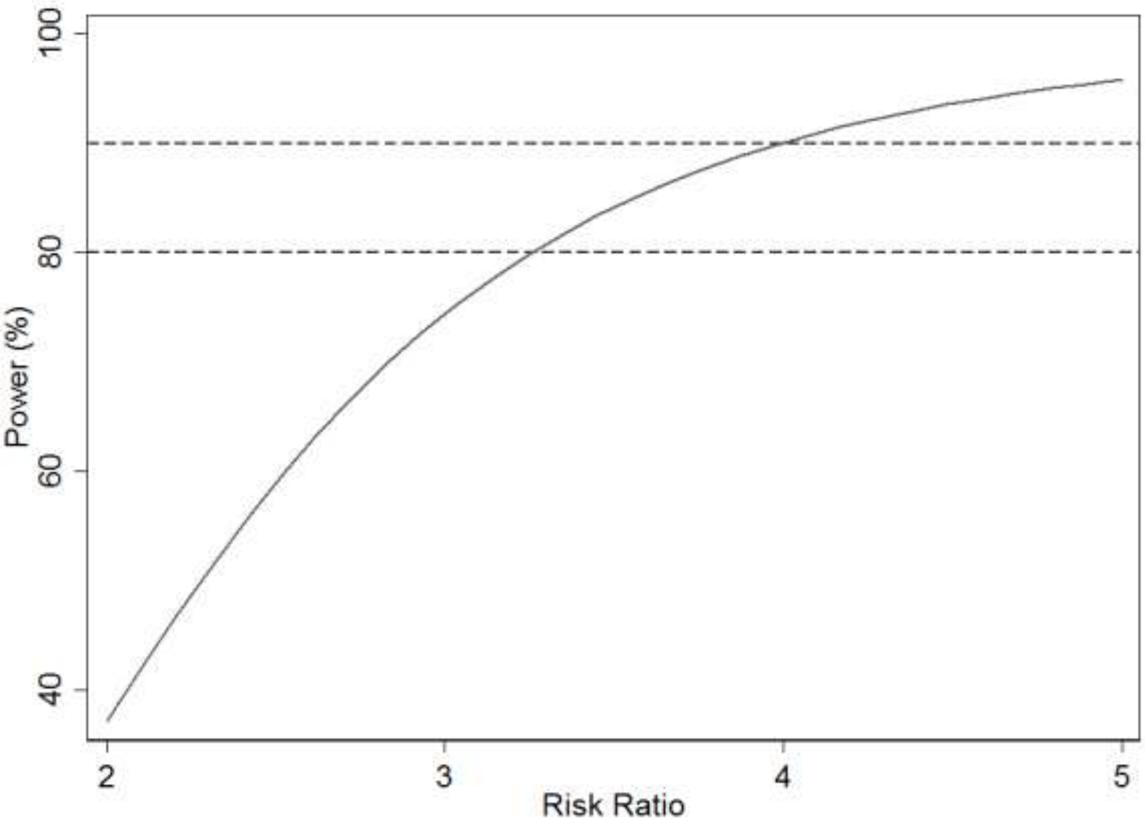

Figure. Power of the study (without attrition) across a range of risk ratios, based on an overall event rate of 2% over two-year follow-up and 20% of patients having CMBs.

The anticipated ICH event rate of 2% over 2 years taking into account attrition suggests that we will observe up to 30 ICH events in total. The 'rule of 10' for developing risk models suggests that this will allow us to develop a risk model with just three predictor variables (45), though more will be possible through use of modern regression techniques (46). It is anticipated that a risk model based solely on CMBs would have a sensitivity of 50% and a specificity of 81% for predicting an ICH within 2 years. A risk model based on more predictors should improve on these values. We expect to use existing summary AF prediction risk scores (incorporating multiple variables, e.g. HAS-BLED) as a single predictor variable to allow us to assess the additional value of including CMBs as a predictor.

ANALYSIS SUMMARY

We plan to compare the rate of ICH between the CMB and CMB-free groups using the log-rank test and will investigate whether the number of CMBs is associated with the risk of ICH using Cox regression. In addition, Cox regression will be used to develop a risk prediction model for ICH. Potential risk factors for the model will be pre-specified in the Statistical Analysis Plan and variable selection methods may be used to reduce the number of predictors in the risk model. Penalised estimation, such as ridge or lasso(46), may be used to guard against over-fitting. Cross-validation, used in conjunction with calibration slopes and the c-index, will be used to internally validate the model and assess calibration, discrimination and predictive accuracy. Missing data, and the reasons for it, will be investigated. Imputation may be used if deemed necessary.

ANALYSIS DETAILS

General Principles

The assumptions underpinning each method will be checked. For example, residuals will be checked for normality where appropriate. The use of transformations or non-parametric methods will be considered if assumptions do not hold. The impact of missing data will be explored in all analyses; sensitivity analyses/multiple imputation will be performed as appropriate. Regression models with interaction terms will be used to perform pre-specified subgroup analyses; the results from these will considered as exploratory because the study is not powered for these. The STROBE guidelines will be followed regarding the reporting of the results of this cohort study.

Flow diagram

A Consort-style flow diagram will be produced to show the numbers of patients:

- Potentially eligible for the study
- Examined for eligibility
- Confirmed eligible
- Included in the study
- Completing each stage of follow-up
- Analysed

Patient Characteristics

Baseline patient characteristics will be described using means (SDs) or medians (interquartile range) for continuous measures, and proportions for categorical measures. These values will be presented by CMB group (with / without) at baseline.

In particular, the following variables will be described (Table 1):
a) Demographic information including age and sex.
b) Clinical information including presence, number and location of CMBs.

These characteristics will be presented separately according to whether CMBs are present at baseline. Confidence intervals and statistical tests (e.g. t-tests and chi-squared tests) will be used as appropriate to investigate whether there are differences between patients with and without CMBs. The number of patients with missing data on variables of interest will also be indicated.

A figure will also be presented showing the number of patients from each centre in the study. This will be broken down CMB status at baseline. The amount of follow-up time available will also be summarised (Table 2).

Outcome data

Study participants will be described with respect to their outcome data.

In particular, the following variables will be described:
a) Number and timings of ICH events.

b) Any stroke, cardiac event, death or major bleeding.

Patients with ICH and without ICH events will be described separately with respect to baseline characteristics (Table 3). Confidence intervals and statistical tests (e.g. t-tests and chi-squared tests, though see below) will be used as appropriate to investigate whether there are differences between patients with and without ICHs. The number of patients with missing data on variables of interest will also be indicated.

Primary Analyses

The primary analysis will be a comparison of the rate of ICH for patients with and without CMBs at baseline (Table 3) using the log-rank test. Assumptions regarding censoring and proportional hazards will be investigated. Non-ICH deaths will be regarded as censoring events though this assumption will be investigated through sensitivity analyses. A chi-squared (or Fisher's exact test) will also be conducted as a sensitivity analysis, assuming that follow-up is reasonably complete.

The primary analysis will use all patients but additional analyses may be performed on those patients that actually received oral coagulation. Adjusted analyses will be carried out using either Cox or logistic regression models as appropriate. Adjustment variables will include age and hypertension measured at baseline (Table 4).

Secondary Analyses

The secondary analyses will be a comparison of the secondary outcomes for patients with and without CMBs at baseline.

Risk Modelling

A risk model that aims to predict the risk of ICH will be developed and validated. The model will be developed using either Cox or logistic Cox regression, depending on the completeness of the follow-up data (Table 5). The risk model will be developed using variables derived from the CMB data, as well as additional variables (measured at baseline). The completeness of a variable will be a factor when considering whether to incorporate it in the regression model.

Due to the anticipated small number of ICH events, care will need to be taken regarding over-fitting of the risk model. Therefore, few predictors will be included in the model (two or three) and shrinkage methods will be used to re-calibrate the model. Variable selection, including pre-screening, many be used if many predictors are available for inclusion in the risk model. Relatively large P-values (e.g. P=0.2) will be used with these procedures. If necessary, penalised regression methods (e.g. lasso) will be used instead of standard regression methods to avoid over-fitting.

Bootstrapping methods will be used to validate the model. Calibration will be assessed using (Miller/Cox/van Houwelingen) calibration slopes. If a Cox model is used, discrimination will be quantified using the (Harrell/Uno) c-index and the D-statistic. If a logistic model is used, discrimination will be quantified using the c-statistic/ROC area and D-statistic, and predictive accuracy will be quantified using the Brier score. The sensitivity, specificity and positive predicted value (PPV) of the (logistic) risk model will also be calculated.

EXAMPLE TABLES

Table 1: Characteristics of patients with and without cerebral microbleeds at baseline

	**With CMB (N =)**	**Without CMB (N =)**	**P-value**
**Clinical Characteristics**			
Mean age in years ± SD (range)			
Female, N (%)			
…			
**Imaging Characteristics**			
Presence (%, range)		n/a	n/a
…		n/a	n/a

Table 2: Available follow-up information on patients

**Information available**	**With CMB (N =)**	**WIthout CMB (N =)**
Baseline only, N (%)		
6 months, N (%)		
12 months, N (%)		
24 months, N (%)		

Table 3: Characteristics of patients with and without intracerebral haemorrhage

	**With ICH (N =)**	**Without ICH (N =)**	**P-value**
**Clinical Characteristics***			
Mean age in years ± SD (range)			
Female, N (%)			
…			
**Imaging Characteristics***			
Presence (%, range)			
…			

* at baseline

Table 4: Cox regression analyses to investigate the association between ICH and the presence of CMBs, adjusted for age and hypertension

	Hazard ratio	95% CI	P-value
Presence of CMBs			
Age (years)			
Hypertension			

Table 5: Cox regression analyses to predict ICH using CMB information and other variables

	Hazard ratio	95% CI	P-value
Presence of CMBs			
…			

* Note: these odds ratios / coefficients may need to be recalibrated / shrunk to optimise predictive abilities

Table 6a: Values of performance measures to assess risk model (Cox)

	Development	Bootstrap adjusted
Calibration		
C-index		
D-statistic		

Table 6b: Values of performance measures to assess risk model (logistic)

	Development	Bootstrap adjusted
Calibration		
ROC area (c-statistic)		
Brier Score		
D-statistic		
Sensitivity*		
Specificity*		
PPV*		

* at cut-point …
